# Communication and Low Mood (CALM): a randomized controlled trial of behavioural therapy for stroke patients with aphasia

**DOI:** 10.1177/0269215512462227

**Published:** 2013-05

**Authors:** Shirley A Thomas, Marion F Walker, Jamie A Macniven, Helen Haworth, Nadina B Lincoln

**Affiliations:** 1 University of Nottingham, Nottingham, UK; 2 Nottingham University Hospitals NHS Trust, Nottingham, UK; 3 Nottinghamshire Health Care Foundation Trust, Nottingham, UK

**Keywords:** Aphasia, mood, depression, behavioural therapy, randomized controlled trial

## Abstract

**Objective:**

The aim was to evaluate behavioural therapy as a treatment for low mood in people with aphasia.

**Design:**

A randomized controlled trial comparing behavioural therapy plus usual care with a usual care control. Potential participants with aphasia after stroke were screened for the presence of low mood. Those who met the criteria and gave consent were randomly allocated.

**Setting:**

Participants were recruited from hospital wards, community rehabilitation, speech and language therapy services and stroke groups.

**Subjects:**

Of 511 people with aphasia identified, 105 had low mood and were recruited.

**Interventions:**

Behavioural therapy was offered for up to three months. Outcomes were assessed three and six months after random allocation.

**Main measures:**

Stroke Aphasic Depression Questionnaire, Visual Analog Mood Scales ‘sad’ item, and Visual Analogue Self-Esteem Scale.

**Results:**

Participants were aged 29 to 94 years (mean 67.0, SD 13.5) and 66 (63%) were men. Regression analysis showed that at three months, when baseline values and communication impairment were controlled for, group allocation was a significant predictor of the Stroke Aphasic Depression Questionnaire (*P* < 0.05), visual analogue ‘sad’ (*P* = 0.03), and Visual Analogue Self-Esteem Scale (*P* < 0.01). At six months, group alone was a significant predictor of the Stroke Aphasic Depression Questionnaire (*P* < 0.05), and remained significant when baseline values were controlled for (*P* = 0.02). Mean Stroke Aphasic Depression Questionnaire 10-item hospital version scores decreased from baseline to six months by six points in the intervention group as compared with an increase of 1.9 points in the control group.

**Conclusions:**

Behavioural therapy seemed to improve the mood of people with aphasia.

## Introduction

Mood disorders are common after stroke (see ref. 1, pp.283–284). They include depression and anxiety, but also general psychological distress, which is not so severe that it leads to a clinical diagnosis but nevertheless has a negative effect on recovery and quality of life. Depression is well recognized following stroke, with an average prevalence of 33%,^2^ and is associated with worse rehabilitation outcomes, increased carer strain, and higher mortality (see ref. 1, p.284). Anxiety disorders also occur in about a third of those with stroke, and post-traumatic stress disorder in between 5% and 30% (see ref. 1, pp.290–291). Patients with aphasia are highly susceptible to depression,^3^ and the severity of communication difficulties may be associated with emotional distress.^4^

Antidepressants may reduce depression, but also increase adverse events,^5^ and are not appropriate for all patients. Some studies have evaluated psychological interventions after stroke, but a Cochrane review reported no evidence of benefit.^5^ One trial found that a brief psychosocial–behavioural intervention plus an antidepressant reduced depression in patients recruited within four months following stroke.^6,7^ Motivational interviewing early after stroke was found to have a beneficial effect on mood at 12 months.^8,9^ However, both studies excluded patients with severe communication difficulties.^6,8,9^ In a trial of cognitive behavioural therapy,^10^ secondary analysis found that the severity of communication difficulties was associated with less improvement in mood,^11^ although only patients with mild communication problems were included.

Most studies of psychological interventions are ‘talk based’ and are not accessible for patients with aphasia. Behavioural therapy is an approach which does not require intact communication and can be adapted for people with aphasia.^12^ Behavioural approaches are based on the behavioural model of depression, in which depression is considered to arise from a lack of positive response contingent social reinforcement from the environment. The aim of therapy is to increase the level of activity, particularly the frequency of pleasant events, in order to improve mood.

Behavioural approaches are effective at treating depression in older adults^13^ and in people with dementia,^14^ and require evaluation in people with aphasia. The aim of this study was to evaluate behavioural therapy for low mood in people with aphasia following stroke.

## Methods

The Communication and Low Mood (CALM) study was a multicentre randomized controlled trial comparing behavioural therapy with usual care (ISRCTN56078830). Ethical approval was obtained from Nottingham Research Ethics Committee 1.

Stroke patients with aphasia were identified on hospital wards, and asked if they were willing to be contacted after discharge from hospital. In addition, referrals were sought from community stroke and rehabilitation services and speech and language therapists. People attending stroke and communication groups in the community were also invited to take part. Potential participants were identified in six centres (Nottingham, Mansfield, Chesterfield, Sheffield, Lincoln and Leicester), between 28 April 2008 and 12 January 2011. Aphasia was confirmed by a speech and language therapist for potential participants recruited through hospital and community services. For potential participants recruited through the voluntary sector, the presence of aphasia was confirmed using the Sheffield Screening Test for Acquired Language Disorders.^15^ People were excluded if they were blind or deaf, had dementia documented in their medical notes, were unable to speak English prior to stroke, or were receiving any treatment for depression at the time of their stroke.

Potential participants were given an information sheet and invited to consent to have their mood screened. Mood was assessed using the ‘sad’ item of the Visual Analog Mood Scales^16^ and the Stroke Aphasic Depression Questionnaire 10-item hospital version,^17^ completed by a nurse, relative or carer. Those who were identified as having low mood on either the visual analogue ‘sad’ item (cut-off >50) or the Stroke Aphasic Depression Questionnaire (cut-off >6)^18^ were then invited to consent to take part in the randomized trial. Informed consent from patients or assent from a relative or carer was obtained. Participants completed further baseline measures, which included measures of language impairment (Sheffield Screening Test), the Frenchay Aphasia Screening Test reading and writing subtests,^19^ Barthel Index,^20^ and a picture-based measure of self-esteem (Visual Analogue Self-Esteem Scale).^21^ Demographic and stroke details were also recorded.

Participants were randomly allocated to one of two groups: behavioural therapy or usual care, using a computer-generated pseudo-random list (1:1 ratio) with permuted blocks of varying sizes, generated by a clinical trials unit with no other involvement in the trial. Randomization was stratified according to recruitment centre and whether the patient was recruited in hospital or in the community. The assistant psychologist providing treatment accessed the allocation by logging into a secure computer server, thus ensuring concealment of allocation.

Patients allocated to either group received all other services that were available to them as local practice.

After randomization, participants allocated to receive behavioural therapy received up to 20 sessions of treatment over three months, with each session lasting approximately 1 hour. Sessions took place at the participant’s place of residence. Therapy was delivered by an assistant psychologist supervised weekly by a clinical psychologist. There was an assistant psychologist based in each of four centres. The additional two centres (Mansfield and Lincoln) were covered by assistants from Chesterfield and Nottingham. In addition, all assistant psychologists attended a joint monthly supervision meeting with a consultant clinical neuropsychologist (JM). The assistant psychologists received training in supported communication from speech and language therapists and were provided with a therapy manual. The manual had been developed from studies of cognitive behavioural therapy for depression after stroke^10^ and with older adults,^22^ and guidelines on conducting cognitive behavioural therapy with people with aphasia.^12^ The intensity of therapy was left to the discretion of the assistant psychologist.

Treatment strategies focused on maximizing mood-elevating activities and included education, activity monitoring, activity scheduling, and graded task assignments. The intervention was tailored to the individual’s needs, and communication resources, such as pictures, photographs and letter charts, were used. The delivery of therapy was monitored by observation of therapy sessions by the chief investigator (ST). The content of therapy was documented using record forms completed by the assistant psychologist after each session.

The primary outcome measure was the Stroke Aphasic Depression Questionnaire 21-item hospital version^17^ at six months after randomization. This scale is an observational measure of mood, which was completed by a relative or carer. This was also completed at three months after randomization. Secondary outcomes were completed by an independent assessor, blind to the participant’s group allocation, at three and six months after randomization. These included the visual analogue ‘sad’ item, Visual Analogue Self-Esteem Scale, and Nottingham Leisure Questionnaire^23^ at three and six months after randomization. In addition, the Carer Strain Index^24^ and patient and carer versions of a 100 mm visual analogue Satisfaction with Care Rating scale (patient and carer versions, adapted from Lincoln et al.^25^) were also completed at six months. The secondary outcome measures were included to assess whether the intervention also improved self-reported mood, leisure activities and carer strain, and these measures are suitable for stroke patients with aphasia. The independent assessor recorded whether the patient was taking antidepressant medication at each outcome assessment.

The sample size estimate was based on the primary outcome measure, the Stroke Aphasic Depression Questionnaire 21-item hospital version, at six-month follow-up. A sample size of 76 in each group was estimated to give 90% power (5% two-sided significance) to detect a difference in mean score of five points on the primary outcome measure, assuming a common standard deviation of 9.42 based on a pilot study (unpublished data) with 22 patients. Allowing for 15% attrition (based on previous stroke rehabilitation studies), the aim was to recruit 176 participants.

Outcomes were analysed by intention to treat. The analysis plan was peer reviewed by two independent stroke experts and an independent statistician before the analysis was conducted. Primary and secondary outcome measures were continuous and analysed using hierarchical multiple linear regression. This approach analyses the effect of continuous and categorical variables on a continuous outcome and has been used in other controlled trials of complex interventions.^26^ For each regression model, the group allocation (behavioural therapy or usual care) was entered in the first block, and baseline values of the outcome variable and Sheffield Screening Test score were entered in the second block. For the Stroke Aphasic Depression Questionnaire model, the baseline score on the 10-item version of the scale was entered in the second block.

The primary analysis was conducted on participants who had complete data for that outcome measure. Sensitivity analyses were conducted to examine the robustness of the primary analysis. Regression models were first repeated including all participants by replacing missing outcome data using the last observation carried forward method,^27^ as the assumption was made that there would be no change in mood or activities over time in the absence of treatment. Second, the regression models were repeated using per protocol data, with participants allocated to receive behavioural therapy included if they received a minimum of three sessions. Outcomes between the intervention and control group were also compared using area under the curve to analyse the differences between repeated measurements,^28^ using Mann–Whitney *U*-tests. A two-sided significance level of 0.05 was used for all analyses. Analyses were performed using SPSS (version 19) for Windows.

## Results

**Figure 1.**
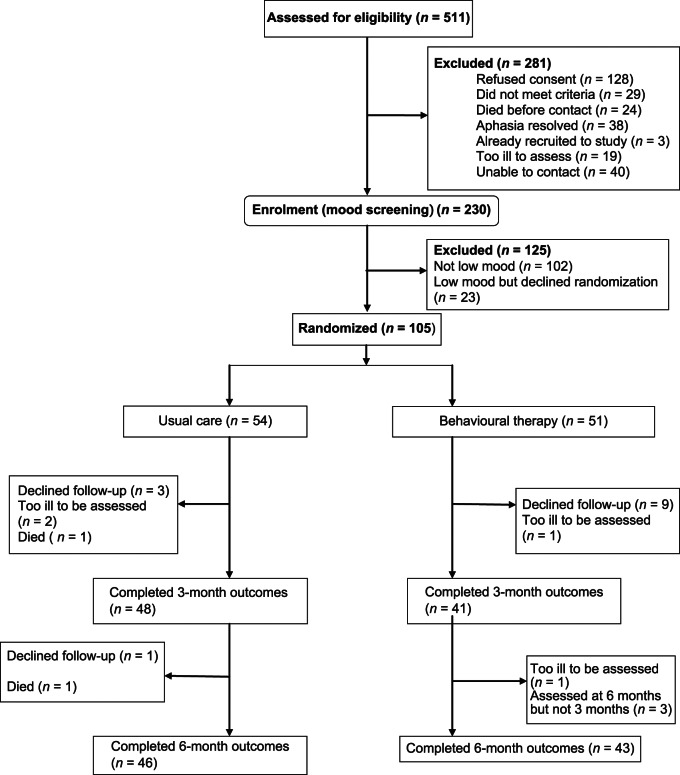
Trial profile.

Of the 511 patients identified, 281 (55%) were excluded and 230 (45%) consented to have their mood assessed. The reasons for exclusion are shown in Figure 1. Of the 230 assessed, 128 (56%) were identified as having low mood and invited to take part in the trial. Of these, 23 refused and 105 were randomized: 54 to usual care and 51 to behavioural therapy. Patient flow through the trial is shown in Figure 1.

Groups were similar on baseline characteristics. Results are shown in Table 1. Patients were predominantly male, married, and living with their spouse; 23 (21%) had had a previous stroke and 29 (28%) were receiving antidepressant medication.

**Table 1.** Baseline demographics and stroke characteristics

Characteristic	Usual care (*n*= 54)		Behavioural therapy (*n* = 51)	
	*n*	%	*n*	%
**Gender** – male	37	69	29	57
**Setting where recruited**				
Hospital	7	13	5	10
Community	47	87	46	90
**Marital status**				
Single	7	13	4	9
Married/partnered	33	61	32	63
Widowed/divorced/separated	14	26	15	29
**Place of residence**				
Independent housing	49	91	49	96
Residential or nursing home	5	9	2	4
**Living arrangements**				
Alone	10	19	13	25
With spouse/partner	33	61	32	63
With relatives	7	13	3	6
Other	4	7	3	6
**Side of lesion**				
Left	38	70	34	67
Right	5	9	3	6
Bilateral	3	6	0	0
Unknown	8	15	14	28
Previous stroke	12	22	11	22
Currently receiving antidepressant medication	14	26	15	29

	Mean	SD	Mean	SD

Age (years)	65.5	13.9	68.5	13.1
Months post stroke (median, interquartile range)	9.0 (4.9–39.0)	–	8.7 (4.1–26.1)	–
Barthel Index (mean, SD)	14.4 (6.0)	6.0	14.8 (4.0)	4.0
**Sheffield Screening Test**				
Receptive	5.5	2.4	5.2	2.5
Expressive	6.2	3.9	6.1	4.1
**Frenchay Aphasia Screening Test**				
Reading	3.6	1.5	3.5	1.7
Writing	2.1	1.9	1.6	1.8
Stroke Aphasic Depression Questionnaire Hospital version 10	9.5	4.4	11.2	5.8
Prorated Stroke Aphasic Depression Questionnaire Hospital version 21	20.1	9.0	23.4	12.2
Visual Analog Mood Scale – ‘sad’	40.6	28.7	48.1	29.0
Visual Analogue Self-Esteem Scale	35.7	8.2	32.8	7.8

Of the 51 patients randomized to behavioural therapy, 48 (94%) agreed to receive therapy and 44 (86%) completed the course of therapy. Patients who completed therapy received a mean of 9.07 sessions (range 3–18, SD 2.63) and the mean duration of each session was 58 minutes (range 30–89, SD 10.71).

At three months and six months after randomization 89 patients completed the outcome assessments. Reasons for non-completion are shown in Figure 1. Complete outcomes were obtained on 85% of participants. Scores on outcome measures are shown in Table 2. The baseline Stroke Aphasic Depression Questionnaire 10-item version scores were prorated to obtain baseline Stroke Aphasic Depression Questionnaire 21-item version scores for the purpose of evaluating progress over time.

**Table 2.** Scores on outcome measures

Measure	Time in months	Usual care			Behavioural therapy		
		*n*	Mean	SD	*n*	Mean	SD
Stroke Aphasic Depression Questionnaire Hospital version 21	3	44	19.2	9.6	39	16.9	10.2
	6	42	21.9	9.5	39	17.4	10.0
Visual Analog Mood Scale – ‘sad’	3	48	36.3	28.4	41	26.5	22.3
	6	46	32.1	29.3	43	25.5	21.5
Visual Analogue Self-Esteem Scale	3	46	33.2	7.4	41	35.4	6.7
	6	44	33.3	7.9	43	34.3	7.3
Nottingham Leisure Questionnaire	3	48	15.7	6.9	41	17.1	6.7
	6	46	15.9	6.8	43	17.0	7.6
Carer Strain Index	6	36	6.3	3.6	37	6.6	3.1
**Satisfaction with Care – Patient**							
Emotional support	6	45	58.0	30.0	41	61.5	31.9
Communication support	6	45	58.2	34.2	41	63.5	31.7
Hospital and community services	6	45	65.3	30.5	41	68.9	26.6
**Satisfaction with Care – Carer**							
Emotional support	6	38	55.1	30.5	37	65.7	27.1
Communication support	6	38	58.6	31.3	37	68.5	28.3
Hospital and community services	6	38	62.8	27.9	37	73.9	21.9

The results of the regression analyses on those who completed outcome measures are shown in Table 3. At three months, group allocation alone was not a significant predictor for any of the outcome measures. When baseline values and communication impairment were controlled for, the effect of allocation became significant for the Stroke Aphasic Depression Questionnaire (*P* = 0.05), visual analogue ‘sad’ item (*P* = 0.03), and Visual Analogue Self-Esteem Scale (*P* = 0.002), but not for the Nottingham Leisure Questionnaire. At six months, group alone was a significant predictor of the primary outcome measure, the Stroke Aphasic Depression Questionnaire (*P* = 0.045), and remained significant when baseline values were controlled for (*P* = 0.022). There was no significant effect of group allocation alone on the visual analogue ‘sad’ item, Visual Analogue Self-Esteem Scale, Nottingham Leisure Questionnaire or Carer Strain Index when baseline values and communication impairment were controlled for.

**Table 3.** Regression analysis of outcome measures for patients with outcome data

	3 months				6 months			
	B	Beta	95% CI	*P*	B	Beta	95% CI	*P*
**Stroke Aphasic Depression Questionnaire Hospital version 21**								
Unadjusted difference	−2.26	−0.12	−6.69 to 2.17	0.313	−4.50	−2.23	−8.89 to −0.11	0.045
Difference adjusted for baseline SADQH−10 and SST	−3.91	−0.20	−7.81 to 0.01	0.050	−6.13	−0.31	−9.95 to −2.30	0.002
**Visual Analog Mood Scale – ‘sad’**								
Unadjusted difference	−9.79	−0.19	−20.65 to 1.07	0.077	−6.58	−0.13	−17.47 to 4.31	0.233
Difference adjusted for baseline VAMS ‘sad’ and SST	−11.65	−0.22	−22.30 to −0.99	0.033	−7.32	−0.14	−18.34 to 3.71	0.190
**Visual Analogue Self-Esteem Scale**								
Unadjusted difference	2.24	0.16	−0.78 to 5.27	0.144	0.96	0.06	−2.31 to 4.24	0.560
Difference adjusted for baseline VASES and SST	3.90	0.28	1.14 to 6.39	0.002	2.51	0.17	−0.39 to 5.40	0.089
**Nottingham Leisure Questionnaire**								
Unadjusted difference	1.14	0.11	−1.44 to 4.30	0.326	1.04	0.07	−1.98 to 4.07	0.495
Difference adjusted for baseline SST	1.57	0.12	−1.23 to 4.40	0.275	1.30	0.09	−1.63 to 4.16	0.387
**Carer Strain Index**								
Unadjusted difference					0.29	0.04	−1.27 to 1.85	0.713
Difference adjusted for baseline SST					0.25	0.04	−1.31 to 1.82	0.749

SADQH-10, Stroke Aphasic Depression Questionnaire 10-item hospital version; SST, Sheffield Screening Test for Acquired Language Disorders; VAMS, Visual Analog Mood Scales; VASES, Visual Analogue Self-Esteem Scale.

Comparison of the area under the curve revealed significant differences between the groups for visual analogue ‘sad’ item (*P* = 0.015), Visual Analogue Self-Esteem Scale (*P* = 0.005) and the Stroke Aphasic Depression Questionnaire (*P* = 0.003) but no significant differences for the Nottingham Leisure Questionnaire (*P* = 0.551).

Sensitivity analysis at three months, replacing missing data using the last observation carried forward on the assumption of no change, showed that, after adjustment for baseline values, group allocation no longer significantly predicted Stroke Aphasic Depression Questionnaire 10-item hospital version (*P* = 0.052), but all other results from three months remained consistent. At six months, the analysis replacing missing data using last observation carried forward showed that group allocation alone remained a significant predictor of Stroke Aphasic Depression Questionnaire scores. After controlling for baseline values, group allocation was now a significant predictor of Visual Analogue Self-Esteem Scale scores (*P* = 0.02) and the visual analogue ‘sad’ item, but the Nottingham Leisure Questionnaire and Carer Strain Index remained non-significant. Per protocol analysis at three and six months showed the same results as the primary intention to treat analysis.

At three months, 15 (28%) of the control group and 11 (22%) of the intervention group were receiving medication for mood problems and this difference was not statistically significant (chi-square = 0.15, *P* = 0.70). At six months, 15 (28%) of the control group and 14 (27%) of the intervention group were receiving medication for mood problems, and this was not significant (chi-square = 0.00, *P* = 1.0).

Satisfaction with Care ratings for both patients and carers were higher for the behavioural therapy group compared with usual care, but these differences were not significant (*P* = 0.06–0.61).

## Discussion

In patients with aphasia and low mood after stroke, allocation to behavioural therapy compared with usual care significantly predicted better self-reported mood, self-esteem, and observer-rated mood three months after randomization. Six months after randomization there was a significant benefit for observer-rated mood. Overall summary scores using the area under the curve showed significant differences in self-reported mood, observer-rated mood, and self-esteem. There was no significant effect of behavioural therapy on leisure activities or carer strain. Both patients and carers in the behavioural therapy group reported higher satisfaction with emotional support, communication support, and hospital and community services, although this did not reach statistical significance.

Behavioural approaches were suitable for patients with aphasia, a group who have previously been largely excluded from studies evaluating psychological treatments for low mood. Differences between the behavioural therapy and usual care groups cannot be attributable to concurrently receiving antidepressant medication, as this was comparable between the groups. Behavioural therapy was shown to have beneficial effects with an average of nine sessions per participant. The optimum intensity and duration of therapy are unknown and it was left to the therapist’s discretion to decide how much treatment to provide. No patients required the maximum 20 sessions allowed, indicating that the intervention did not need to be delivered as intensively as expected. However, the three-month intervention period did not allow flexibility to provide follow-up therapy visits to support the maintenance of benefits, as might be provided in a clinical service.

Mitchell et al.^6^ found benefit at 12 months’ follow-up of a psychosocial–behavioural intervention given for only eight weeks early after stroke, although patients with receptive or global aphasia were excluded. It is possible that a longer duration than the three months provided in this study may be required for some patients to sustain the early benefits of therapy, but exploration of patient characteristics affecting outcome would be needed to inform changes to therapy intensity and duration. As we did not include an attention control group, we cannot draw conclusions about the ‘active ingredients’ of therapy or the role of non-specific attention. As noted by Watkins et al.,^9^ it is difficult to identify an appropriate attention control. This next stage is required in order to ascertain the essential components of the therapy provided.

The absence of evidence of benefit on leisure activities may reflect that the Nottingham Leisure Questionnaire was not sensitive enough to detect changes in leisure activities following therapy. As the Nottingham Leisure Questionnaire was not completed at baseline it was not possible to assess improvement from before the intervention. Also, it may not only be the level of leisure activities themselves that is relevant, but the value that the patient places on activities that they are able or unable to take part in.

The generalizability of the findings may be suggested by the fact that that the trial was multicentre, open to patients with any severity of aphasia, and followed a therapy manual, and the intervention was delivered by multiple therapists, who reflected the experience level of NHS assistant psychologists who could deliver the therapy in clinical practice under the supervision of a clinical psychologist. There was no restriction on how long ago the patients had had their stroke, while other studies of psychological interventions have recruited patients within 28 days,^8,9^ two months^29^ and four months^6^ after stroke. Unlike single centre studies, the results cannot be attributable to a specific therapist or local setting, although we cannot be sure of the comparability of usual care across study sites. The randomization was stratified by study site, so any differences in usual care would not have masked any benefits of therapy. Acceptability of the intervention to participants was not evaluated. The content of the therapy varied between participants, and therefore it is difficult to define the intervention precisely. However, this reflects clinical practice, and indicates that the overall approach is useful rather than any specific techniques.

Participants were recruited from a variety of sources at a wide range of times since stroke and are likely to be representative of those who would be referred in clinical practice. However, in clinical practice, patients would be referred for behavioural therapy based on the severity of mood problems and suitability for the intervention, and therefore some people may have been included whom clinical therapists would not have expected to benefit. The recruitment rate was lower than planned, and so the intended sample size was not achieved in the study recruitment period due to the time taken to identify and visit potential participants from a variety of sources. Future studies should increase the number of recruiting centres. The rate of low mood was 60%, which is high, but probably reflects the fact that participants were being recruited for a treatment study and so the referring clinicians may have been biased to identifying potential participants with low mood.

Few assessments are available to screen for mood problems in patients with aphasia. The Visual Analog Mood Scales ‘sad’ item and Stroke Aphasic Depression Questionnaire were used, with patients meeting the criteria on one or both measures to be eligible. The use of these criteria may have led to under-representation of patients with severe aphasia, and some patients with low mood may have been missed. The mean scores on the ‘sad’ item were below the cut-off of 50, indicating that the sample included some patients with no self-reported severe mood problems, although mood did still improve at three months.

There are limitations to both observer-rated and visual analogue scales to assess mood in those with communication problems, and therefore the strategy was to consider low mood as reflected in either type of measure. In addition, the Stroke Aphasic Depression Questionnaire 10-item hospital version was used at baseline, whereas the 21-item version of the scale was used at follow-up. This occurred because the 10-item version was in routine clinical use on the wards where patients were recruited, whereas the 21-item version has been shown to be more sensitive and therefore was considered a more appropriate outcome measure. An additional problem of the Stroke Aphasic Depression Questionnaire at outcome was that the relative or carer completing the scale knew whether the participant had received treatment. However, as the results are consistent between observer-rated and independently assessed measures, this suggests the findings are not simply due to observer bias.

The study was limited by the small sample size, which may not have had adequate power to detect significant effects across all analyses. The differences in leisure activities and satisfaction were in the predicted direction, but the study may not have had sufficient power to detect significant differences on these measures. However, the findings are promising. Future studies of behavioural therapy should recruit a larger sample size, identify patients with more severe mood problems, and investigate whether treatment duration should be extended to facilitate the maintenance of treatment gains. Overall the results of this preliminary study suggest that behavioural therapy improved the mood of stroke patients with aphasia and low mood. Further evaluation of this treatment strategy is therefore warranted.

**Clinical messages**

- Mood problems are common in people with aphasia.
- Behavioural treatments are appropriate for those with low mood and communication problems.
- In a randomized trial, behavioural therapy for an average of 10 sessions improved participants’ mood.

## References

[1] Lincoln NB Kneebone II Macniven JAB Morris R Psychological Management of Stroke. Oxford: Wiley-Blackwell, 2012

[2] Hackett ML Yapa C Parag V Anderson CS Frequency of depression after stroke: a systematic review of observational studies. Stroke 2005; 36: 1330–1340 15879342 10.1161/01.STR.0000165928.19135.35

[3] Kauhanen ML Korpelainen JT Hiltunen P Aphasia, depression, and non-verbal cognitive impairment in ischaemic stroke. Cerebrovasc Dis 2000; 10: 455–461 11070376 10.1159/000016107

[4] Thomas SA Lincoln NB Predictors of emotional distress after stroke. Stroke 2008; 39: 1240–1245 18292381 10.1161/STROKEAHA.107.498279

[5] Hackett ML Anderson CS House A Xia J Interventions for treating depression after stroke. Cochrane Database Syst Rev 2008:CD003437 18843644 10.1002/14651858.CD003437.pub3

[6] Mitchell PH Veith RC Becker KJ Brief psychosocial-behavioral intervention with antidepressant reduces poststroke depression significantly more than usual care with antidepressant: living well with stroke: randomized, controlled trial. Stroke 2009; 40: 3073–3078 19661478 10.1161/STROKEAHA.109.549808 PMC2777736

[7] Mitchell PH Teri L Veith R Living well with stroke: design and methods for a randomized controlled trial of a psychosocial behavioral intervention for poststroke depression. J Stroke Cerebrovasc Dis 2008; 17: 109–115 18436150 10.1016/j.jstrokecerebrovasdis.2007.12.002 PMC2396193

[8] Watkins CL Wathan JV Leathley MJ The 12-month effects of early motivational interviewing after acute stroke: a randomized controlled trial. Stroke 2011; 42: 1956–1961 21700946 10.1161/STROKEAHA.110.602227

[9] Watkins CL Auton MF Deans CF Motivational interviewing early after acute stroke: a randomized, controlled trial. Stroke 2007; 38: 1004–1009 17303766 10.1161/01.STR.0000258114.28006.d7

[10] Lincoln NB Flannaghan T Cognitive behavioral psychotherapy for depression following stroke: a randomized controlled trial. Stroke 2003; 34: 111–115 12511760 10.1161/01.str.0000044167.44670.55

[11] Thomas SA Lincoln NB Factors relating to depression after stroke. Br J Clin Psychol 2006; 45: 49–61 16480566 10.1348/014466505X34183

[12] Grober S Hibbard MR Gordon WA Stein PN Freeman A The psychotherapeutic treatment of post-stroke depression with cognitive behavioural therapy. In: Gordon WA (ed.) Advances in Stroke Rehabilitation. Andover: Andover Medical Publishers, 1993, pp. 215–241

[13] Pinquarti M Duberstein PR Lyness JM Effects of psychotherapy and other behavioural interventions on clinically depressed older adults: a meta-analysis. Aging Ment Health 2007; 11: 645–657 18074252 10.1080/13607860701529635

[14] Teri L Logsdon RG Uomoto J McCurry SM Behavioral treatment of depression in dementia patients: a controlled clinical trial. J Gerontol B Psychol Sci Soc Sci 1997; 52: 159–166 10.1093/geronb/52b.4.p159 9224439

[15] Syder D Body R Parker M Boddy M Sheffield screening test for acquired language disorders. Windsor, UK: NFER-Nelson, 1993

[16] Stern RA Visual Analog Mood Scales professional manual. Odessa, FL: Psychological Assessment Resources Inc, 1997

[17] Lincoln NB Sutcliffe LM Unsworth G Validation of the Stroke Aphasic Depression Questionnaire (SADQ) for use with patients in hospital. Clinical Neuropsychological Assessment 2000; 1: 88–96

[18] Bennett HE Thomas SA Austen R Morris AMS Lincoln NB Validation of screening measures for assessing mood in stroke patients. Br J Clin Psychol 2006; 45: 367–376 17147102 10.1348/014466505x58277

[19] Enderby P Wood V Wade D Frenchay Aphasia Screening Test. Oxford: Whurr Publishers, 1997

[20] Collin C Wade DT Davies S Horne V The Barthel Index: a reliability study. Int Disabil Stud 1988; 10: 61–63 3403500 10.3109/09638288809164103

[21] Brumfitt S Sheeran P Vases: Visual Analogue Self-Esteem Scale. Bicester: Winslow Press Ltd, 1999 10.1348/014466599162980 10590826

[22] Laidlaw K Knight BG Handbook of emotional disorders in later life: Assessment and treatment. Oxford:Oxford University Press, 2008

[23] Drummond AER Walker MF The Nottingham Leisure Questionnaire for stroke patients. Br J Occup Ther 1994; 57: 414–418

[24] Robinson B Validation of a Caregiver Strain Index. J Gerontol 1983; 38: 344–348 6841931 10.1093/geronj/38.3.344

[25] Lincoln NB Francis VM Lilley SA Sharma JC Summerfield M Evaluation of a stroke family support organiser: a randomized controlled trial. Stroke 2003; 34: 116–121 12511761 10.1161/01.str.0000047850.33686.32

[26] Lincoln NB Yuill F Holmes J Evaluation of an adjustment group for people with multiple sclerosis: a randomized controlled trial. Mult Scler 2011; 17: 1250–1257 21613332 10.1177/1352458511408753

[27] Streiner D Geddes J Intention to treat analysis in clinical trials when there are missing data. Evid Based Ment Health 2001; 4: 70–71 12004740 10.1136/ebmh.4.3.70

[28] Matthews JN Altman DG Campbell MJ Royston P Analysis of serial measurements in medical research. BMJ 1990; 300: 230–235 2106931 10.1136/bmj.300.6719.230 PMC1662068

[29] Williams LS Kroenke K Bakas T Care management of post-stroke depression: a randomized controlled trial. Stroke 2007; 38: 998–1003 17303771 10.1161/01.STR.0000257319.14023.61

